# The Evolution of Mitral Valve Surgery: the Future in the Hand of Robots

**DOI:** 10.21470/1678-9741-2019-0192

**Published:** 2020

**Authors:** Amer Harky, Hiu Tat Kwok, Ka Siu Fan

**Affiliations:** 1 Department of Cardiothoracic Surgery, Liverpool Heart and Chest, Liverpool, UK.; 2 Leicester Medical School, Leicester, UK.; 3 St. George’s, University of London, London, UK.

**Keywords:** Mitral Valve, Sternotomy, Robotics, Length of Stay, Robotic Surgical Procedures, Cardiac Surgical Procedures, Minimally Invasive Surgical Procedures

## Abstract

**Objective:**

To examine the current literature behind the evolution of mitral valve surgery techniques and their impact on patient outcomes.

**Methods:**

An electronic literature search among major databases was performed (PubMed, Embase, Scopus, Cochrane, and Google scholar). All the relevant articles were screened and identified to be included in this narrative review. The main outcomes were postoperative morbidity, length of in-hospital stay, and long-term mortality.

**Results:**

Minimally invasive and robot-assisted approach to mitral valve repair and replacements has shown great potential in improving surgical outcomes when compared against traditional midline sternotomy. Selected patients can benefit from percutaneous mitral valve surgery; however, more evidence is required to ascertain its long-term outcomes.

**Conclusion:**

Current evidence suggests that robotic and minimal invasive mitral valve surgeries are increasing in practice with satisfactory perioperative and mortality rates. However, long-term data is yet to be published to support current practice.

**Table:** 

Abbreviations, acronyms & symbols				
ACx	= Aortic cross-clamp		MR	= Mitral regurgitation
AF	= Atrial fibrillation		MS	= Mitral stenosis
CABG	= Coronary artery bypass graft		MVr	= Mitral valve repair
CI	= Confidence interval		MVR	= Mitral valve replacement
CPB	= Cardiopulmonary bypass		NYHA	= New York Heart Association
FDA	= Food and Drug Administration		OR	= Odds ratio
HR	= Hazard ratio		RA	= Right atrium
ICS	= Intercostal space		RCT	= Randomized controlled trial
ICU	= Intensive care unit		ROB	= Robotic approach
LA	= Left atrium/atrial		RR	= Relative risk
LOS	= Length of in-hospital stay		SMD	= Standardised mean difference
LV	= Left ventricle		SMR	= Standardised mortality ratio
LVESVI	= Left ventricular end systolic volume index		TIA	= Transient ischemic attack
MIMVR	= Minimally invasive mitral valve repair		TS	= Transseptal
MIMVS	= Minimally invasive mitral valve surgery		WMD	= Weighted mean difference

## INTRODUCTION

Valvular heart diseases represent a growing public health problem; it is becoming increasingly prevalent among our ageing population and affects up to 2.5% of the general population^[1]^. Most of these diseases are of the mitral valve, such as stenosis, regurgitation, and prolapse. The mainstay management is surgical intervention, which classically relies on sternotomy; this provides access to mediastinum and allows direct visualization of the valves. While it may have been our only option for many years, it is incredibly traumatic and not without risks. Various alternative approaches have since been developed to provide access, namely right lateral mini-thoracotomy, robot-assisted mitral surgery, and percutaneous mitral surgery. In this review, we will examine the current evidence behind each surgical approach and their potential in improving patient care in the future.

Milton first proposed the mid-sternotomy approach in 1897, but it was not widely used until Julian et al.^[2]^ reintroduced it in 1957. This technique remains the key access incision for most of cardiac surgeries due to a broad field of exposure and accessibility to the heart and the great vessels^[2]^. However, in 1996, Carpentier et al.^[3]^ demonstrated the first minimally invasive mitral valve surgery (MIMVS) through mini-thoracotomy. Reser et al.^[4]^ suggested a right lateral mini-thoracotomy approach in which a 6 cm incision is made over the fifth intercostal space (ICS) in the inframammary groove, in men, or the sub-mammary crease, in women, extending from the anterior to medial axillary line to minimize the visibility of the postoperative scar. This technique has evolved dramatically, especially in patients that require mitral valve repair (MVr), and very good results have been reported in the literature^[5,6]^.

Since the invention of the da Vinci robotics system in 1998, its application in various surgical specialties quickly grew. MVr is now the most commonly performed robot-assisted cardiac surgery and is able to overcome many drawbacks of the traditional sternotomy^[7]^. This is achieved by providing access through incisions of only a few centimeters to access the mediastinum and the heart. Robotic assistance also augments surgeon’s technical skills by providing superior instrument articulation, tremor filtering, and motion scaling^[8]^. The greatly reduced trauma benefits patients by improving both recovery speed and complication rates. Current literature examining the robotic approach commonly examine outcome parameters such as mortality, stroke, atrial fibrillation (AF), length of in-hospital stays (LOS), aortic cross-clamp (ACx) time, and cardiopulmonary bypass (CPB) time. They generally reflect comparable success rates and mortality with the benefits of reduced complication rates and LOS. Despite the advantages, many studies also reported increased ACx and CPB times, which may predict postoperative complications and mortality^[9]^.

MitraClip was first implanted in 2003 in Vienna and later approved by the Food and Drug Administration (FDA) in 2013^[10]^. It is a percutaneous edge-to-edge repair system for transseptal (TS) mitral valve surgery. A catheter-delivered clip is delivered and forms a double orifice to reduce the regurgitation jet. Other devices for transcatheter MVr were also developed and trialled on humans, recently including CardiAQ®, Tiara®, FORTIS®, and Tendyne®^[11]^.

### To Repair or Replace the Diseased Mitral Valve

MVr or mitral valve replacement (MVR) is indicated when there is severe mitral valve disease, and the repair is mostly used in cases of regurgitation. The indications for MVr and MVR are largely reported and can be different in each category. Mitral stenosis (MS) happens when the valve leaflets become thickened, calcified, and immobile. This leads to obstruction in blood flow from the left atrium (LA) to the left ventricle (LV). Common causes of MS include rheumatic valve disease through commissural fusion, chordal shortening, and fusion^[12]^. It can be graded as mild, moderate, and severe, depending on parameters such as valve area, mean gradient, and pulmonary artery pressure. Severe MS is defined as valve area of < 1.0 cm^2^, with supporting findings including mean gradient of < 10 mmHg, and pulmonary artery pressure of > 50 mmHg, according to the American Society of Echocardiography^[13]^.

There are qualitative and quantitative criteria for severe mitral regurgitation (MR). Qualitative criteria includes angiographic grade of 3+, which indicates that the density of contrast in the atrium and ventricle equalize after several beats, and of 4+, which shows the LA becomes as dense as the LV on the first beat and contrast is seen refluxing into the pulmonary veins^[14]^, and color Doppler jet area with vena contracta width > 0.7 cm, with large central MR jet (area > 40% of LA area), or with a wall-impinging jet of any size, swirling in LA. Quantitative criteria include regurgitation volume of ≥ 60 ml per beat, regurgitation fraction of ≥ 50%, and regurgitation orifice area of ≥ 0.40 cm^2^.

The New York Heart Association (NYHA) functional classification is a common way to evaluate symptoms of heart failure, namely the patients’ exercise tolerance. Class III is defined as marked limitation of physical activity, where less than ordinary activity causes fatigue, palpitation, or dyspnea, but the patient remains comfortable at rest. Class IV is defined as being unable to carry on any physical activity without discomfort and the patient experiences symptoms of heart failure while at rest. If any physical activity is undertaken, discomfort increases^[15]^. However, without a consistent method for assessing NYHA grade, the inter-operative study on Class II and III patients showed poor concordance when used by independent cardiologists^[16]^.

Studies have compared the safety and efficiency of the two approaches for treating degenerative MR. A meta-analysis by Jung et al.^[17]^ analysed 12 retrospective studies which included 2,950 MVr and 1,252 MVR patients with degenerative MR^[17]^. It showed that the MVR group has a higher mortality than the MVr group (hazard ratio [HR] 1.57; 95% confidence interval [CI] 1.39-1.77; *P*=0.81). MVR is also associated with a higher risk of reoperation (HR 1.47; 95% CI 1.09-1.98) than MVr. However, there are several limitations to this study. The observed patterns may be attributable to selection bias as these MVR patients tend to have multiple comorbidities. For the most accurate comparison, patients should ideally be paired with others with similar comorbidities and backgrounds.

A study by Acker et al.^[18]^ compared MVr against MVR for severe ischaemic MR cases. Two hundred fifty-one patients were recruited and assigned to either MVr or chordal-sparing MVR. The left ventricular end systolic volume index (LVESVI), a parameter reflective of LV function and predictor of postoperative status, was measured at 12 months after the procedure. The results showed that the mortality rate of MVr group and MVR group are 14.3% and 17.6%, respectively, and that there was no significant difference (HR 0.79; 95% CI 0.42-1.47; *P*=0.45). However, the rate of recurrence of MR was higher in the MVr group (32.6% *vs*. 2.3%, respectively; *P*<0.001).

A meta-analysis by Salmasi et al.^[19]^ looked at 11 studies to determine the outcomes for moderate ischaemic MR. Eight hundred sixty-four patients had coronary artery bypass graft (CABG) and 542 patients had CABG and MVr. The results showed that there was no significant difference in operative mortality (odds ratio [OR] 1.56; 95% CI 0.92‐2.71; *P*>0.05) and long-term survival (HR 0.98; 95% CI 0.71-1.35; *P*>0.05) rates. Patients who had both CABG and MVr improved their MR grade significantly (weighted mean difference [WMD] -1.15; 95% CI -1.67 to -0.064; *P*<0.001) and their left ventricular systolic diameter (WMD -3.02; 95% CI -4.85 to -1.18; *P*=0.001)^[19]^. It showed that MVr can be beneficial as an add-on procedure to improve the surgical outcome. However, results must be interpreted with caution, since it examines concurrent MVr in CABG surgeries, whose intervention and comorbidities may affect the outcomes.

Harky et al.^[20]^ reported satisfactory outcomes of mitral valve repair in patients with native valve endocarditis when compared to valve replacement.

Table 1 summarizes the important findings for five studies which compared MVr and MVR. The results for MVr are consistent across the studies and showed better long-term survival comparing to MVR.

**Table 1:** Summary of studies comparing mitral valve repair (MVr) and mitral valve replacement (MVR).

Author	Population (n)		Mortality at 30 days (%)		Mortality at 12 months (%)		Important findings
	MVr	MVR	MVr	MVR	MVr	MVR	
Acker et al.^[18]^	126	125	1.6	4.0	14.3	17.6	Rate of recurrence was higher in the MVr group
Gillinov et al.^[42]^	447	232	3.1	5.6	8	12	MVr shows a survival advantage after 2 years
Suri et al.^[43]^	1173	238	0.7	5.6	N/A	N/A	MVr shows better long-term survival
Zhou et al.^[44]^	241	78	2.5	9	6	19.6	MVr is a better treatment for degenerative mitral regurgitation
Daneshmand et al.^[45]^	705	284	2.3	3.5	N/A	N/A	MVr links to better survival after 10-15 years

### Minimal Access or Full Sternotomy

The minimal access of mitral surgery was performed through either partial sternotomy approach or mini-thoracotomy approach.

A meta-analysis by Cao et al.^[6]^ compared the outcomes of full sternotomy against minimal invasive techniques from seven studies, including one randomized controlled trial and six retrospective studies. Milton first proposed the mid-sternotomy approach in 1897, but it was not widely used until Julian et al.^[2]^ reintroduced it in 1957. Its measured endpoints included mortality rate, sternal wound infection, cardiovascular comorbidities, renal failure, reoperation for bleeding, readmission within 30 days, ACx time, CPB time, and LOS, including intensive care unit (ICU) length of stay.

For mortality, 952 cases of minimally invasive mitral valve repair (MIMVR) and 1,011 cases of sternotomy were identified, and it showed that there is no significant difference between the two approaches (relative risk [RR] 1.23; CI 0.22-6.88; *P*=0.81). Statistical analysis for risk of sternal wound infection, cardiovascular comorbidities, renal failure, reoperation for bleeding, and readmission within 30 days also showed no significant difference (all *P*>0.05).

Patients who had MIMVR spent less time in the ICU (standardised mortality ratio [SMR] -0.77; 95% CI -1.36 to -0.17; *P*=0.01), but the procedure was associated with longer ACx time (SMR 1.47; 95% CI 0.52-2.42; *P*=0.003). Al-Sarraf et al.^[21]^ reported in their study about the prolonged ACx time in 3,799 patients. The study showed that prolonged ACx time is associated with lower cardiac output, prolonged ventilation time, higher renal complications, prolonged LOS, blood transfusion, and increased mortality. Similarly, Cao et al.^[6]^ also identified a significantly longer CPB time in MIMVR cases. A study by Adamik et al.^[22]^ showed that prolonged CPB time is associated with intestinal ischaemic damage and endotoxaemia from ischaemic-reperfusion injury. Thirty-four patients were studied, and it showed that prolonged CPB time increases the level of intestinal fatty acid-binding protein, a biomarker that may indicate intestinal damage. This can lead to translocation of bacteria and endotoxin, which can cause sepsis. Although the study was performed on patients who had CABG, the risk of ischaemic-reperfusion injury should still be considered for mitral valve surgery since CPB is utilized in both operations.

Echocardiography outcomes were measured in both groups of patients and results remained similar. Preoperatively, 98.7% of the patients who underwent MIMVR had moderate or severe MR compared to 98.4% for the full sternotomy group. Postoperatively, 99.7% of patient who had MIMVR had none/trivial/mild MR compared to 99.6% for the sternotomy group.

Table 2 is a summary of the key studies that compared MIMVR *vs*. full sternotomy mitral valve intervention. While MIMVR is associated with less transfusion, it is often accompanied with prolonged ACx and CPB times. With evidence that CPB time may affect the surgical outcome, it is important to explore its role within mitral valve surgery when deciding on the mainstay treatment approach. Despite such concerns, its postoperative results are generally similar to the sternotomy results; with patients subjected to fewer traumas and reducing their LOS, MIMVR may eventually prove to be the superior approach.

**Table 2:** Summary of studies comparing minimally invasive mitral valve repair (MIMVR) and sternotomy.

Author	Population (n)		Mortality (%)		Important findings
	MIMVR	Sternotomy	MIMVR	Sternotomy	
Grossi et al.^[46]^	100	100	0	1	MIMVR is linked to less plasma transfusion, fewer postoperative complication, and shorter LOS
Mihaljevic et al.^[47]^	474	337	0.21	0.30	MIMVR results are equal to or better than sternotomy results
Ryan et al.^[48]^	117	117	0	0	MIMVR links to reduction in ICU time, ventilation time, and LOS with no increase in morbidity
Suri et al.^[49]^	350	365	0.57	0	MIMVR has longer ACx and CPB times, but early outcomes are similar to the sternotomy ones
Goldstone et al.^[5]^	153	153	0	0	Right mini-thoracotomy approach does not compromise clinical outcomes

ACx=aortic cross-clamp; CPB=cardiopulmonary bypass; ICU=intensive care unit; LOS=length of in-hospital stay

### Robot-Assisted Mitral Surgery

With robotic MVr being the least invasive approach available, it is expected that the outcomes are superior to traditional sternotomy or thoracotomy. Robot-assisted MVr is normally performed through a 2 cm lateral working port or a 3-4 cm right anterolateral mini-thoracotomy^[7]^. In addition, this can achieve six degrees of freedom, rather than the four provided by typical endoscopic instruments, and allow wrist-like motions^[7]^. Through combining high-resolution, magnified 3-dimensional images with excellent hand-eye coordination systems, the robotics system delivers safe and effective repairs. By eliminating the need for traditional sternotomy, patients experience less pain, quicker recovery, and lower risks^[23,24]^.

However, there is a limited amount of literature, of adequate sizes, that directly compare the robotic and the traditional approach. In a study of 759 posterior mitral valve prolapse repairs, Mihaljevic et al.^[25]^ compared the robotic approach with full sternotomies, partial sternotomies, as well as right mini-anterolateral thoracotomies; they found no statistically significant differences in quality of repair (*P*=0.6, 0.2, and 0.1, respectively). While neurologic, pulmonary, and renal complications were similar (*P*>0.1), the robotic group had the lowest occurrence of AF and pleural effusion, which contributed to reduced LOS. Prevalence of AF in the robotic group was lower by 4% (*P*=0.5), 13% (*P*=0.002), and 7% (*P*=0.3) when compared to full sternotomy, partial sternotomy, and mini-anterolateral thoracotomy, respectively, while pleural effusion was reduced from 8.5% to 0% (*P*=0.002) and from 8.5% to 1.8% (*P*=0.001) when compared with full sternotomy and partial sternotomy, respectively. LOS were reduced by 1.0, 1.6, and 0.9 days (all *P*<0.001) when compared to full sternotomy, partial sternotomy, and mini-anterolateral thoracotomy, respectively. However, it should be noted that the robotic group also had the longest operative times, median of 387 minutes, and it is 109, 110, and 60 minutes longer than the full sternotomy, partial sternotomy, and mini-anterolateral thoracotomy groups (all *P*<0.0001), respectively. A study of 745 mitral valve prolapse repairs also identified longer ACx times (75 *vs*. 35 minutes, *P*<0.001) and perfusion time (101 *vs*. 40 minutes, *P*<0.001) in the robotic group^[26]^. Similarly, postoperative outcomes (*P*=1.00) and AF incidence (*P*=0.60) remained statistically insignificant^[26]^.

Similarly, a meta-analysis of 1,650 patients, across six retrospective studies, demonstrated that the robotic approach improved perioperative outcomes over the traditional sternotomy^[27]^. The operations within the studies were all performed through two or three ports and used the da Vinci surgical system. Mortality rates were reported by two of the studies and the robotic approach showed significant benefits of 0.5% *vs*. 2.2% (RR, 0.32; 95% CI 0.12-0.83; *P*=0.02)^[27]^. Of the three studies that reported incidence of perioperative stroke, no statistically significant differences were observed: 0.8% vs. 2.4% (RR, 0.50; 95% CI 0.05-4.65; *P*=0.54). Re-operation did not differ across all studies: 3.0% *vs*. 3.7% (RR, 0.82; 95% CI 0.47-1.42; *P*=0.47). As with the increased operative times seen in the previous study, the ACx and CPB times were significantly longer in the robotic surgery: standardised mean difference (SMD) were 2.05 (95% CI 1.23-2.87; *P*<0.00001) and 3.03 (95% CI 0.84-5.23; *P*<0.007). Contrarily, a calculated SMD of -1.07 (95% CI -2.83 to -0.7; *P*=0.24) in LOS is not significantly different between the approaches. However, it is important to note that mortality differences were no longer significant when the largest study was removed, suggesting that validities of these effects are yet to be fully substantiated.

While promising, the meta-analysis must be interpreted with caution as only retrospective studies were included and no randomized study was involved, which means that the results may reflect an unbalanced patient baseline characteristic instead. Cao et al.^[27]^ concluded that with the lack of large randomized trials, current observed benefits may be the result of heterogeneous patient cohort between the two treatment arms. As demonstrated, the current literature is not adequate to draw accurate conclusions over benefits and comorbidities of the robotic approach; hence, large randomized studies should be conducted. Table 3 is a summary of key studies in robotic MVr.

**Table 3:** Summary of studies on robot-assisted mitral valve surgeries.

Author	Population (n)		Mortality rates (%)		Stroke incidence (%)		AF incidence (%)		LOS (days)		Important findings
	ROB	Sternotomy	ROB	Sternotomy	ROB	Sternotomy	ROB	Sternotomy	ROB	Sternotomy	
Stevens et al.^[50]^	447	377	1.1	3.8	0.7	3.4	28	26	04-Jun	05-Aug	ROB is associated with reduced neurologic events but longer CPB and ACx times
Mihaljevic et al.^[25]^	261	498*	0	0	1.8-2.7**	0-3.1**	19-26**	26-35**	4.2	5.2-5.8**	ROB is as safe as the traditional approach, offers shorter LOS, and is less invasive
Suri et al.^[26]^	197	294	0	0	1.05	0	20	23	4.46	5.34	ROB offers effective correction of all categories of valve prolapse with shorter LOS and little adverse events
Woo et al.^[51]^	25	71	0	1.4	N/A	N/A	N/A	N/A	7.1	10.6	ROB offers a minimally invasive approach, shorter hospitalization, and reduced need for blood transfusion
Kam et al.^[52]^	107	40	0	0	N/A	N/A	N/A	N/A	6.47	8.76	ROB can be performed with similar success rates and costs, but has slightly longer operative time
Folliguet et al.^[53]^	25	25	0	0	4***	8***	N/A	N/A	7	9	ROB is comparable to sternotomy, but long-term follow-up is needed to determine durability of the repair
Suri et al.^[54]^	487	N/A	0.2	N/A	0.8	N/A	N/A	N/A	3	N/A	ROB offers excellent survival rates with infrequent complications regardless of repair complexity
Murphy et al.^[55]^	1257	N/A	0.9	N/A	0.7	N/A	13.2	N/A	4.9±4.4	N/A	ROB surgery, including concomitant procedures, is safe and effective
Ramzy et al.^[56]^	300	N/A	0.3	N/A	1.7	N/A	5.7	N/A	6.0±2.9	N/A	ROB is effective, but presents a significant learning curve, and sustained training is required to stay proficient and ro reduce operating time
Tatooles et al.^[57]^	25	N/A	0	N/A	0	N/A	20	N/A	2.68	N/A	While ROB can be performed, long-term follow-up is needed to determine durability of the repair

ACx=aortic cross-clamp; AF=atrial fibrillation; CPB=cardiopulmonary bypass; LOS=length of in-hospital stay; ROB=robotic approach

* Includes complete sternotomy, partial sternotomy, and right mini-anterolateral thoracotomy.

** Results of matched pairs between robotic and both complete and partial sternotomies.

*** Only transient ischaemic attacks, not strokes, were recorded.

### Percutaneous Mitral Valve Intervention

Despite a successful trial by Feldman et al.^[28]^ in the Endovascular Valve Edge-to-Edge Repair Study, the FDA initiated a Class I recall for the MitraClip Delivery System in 2016^[28]^. Due to malfunction, some delivery systems could not be detached from the clip and lead to subsequent open-heart surgeries to retrieve the devices^[29]^. Recently, MitraClip is licensed for patients with normal mitral valves who develop heart failure and MR after unsatisfactory response from optimal medical therapy^[30]^. Many devices were developed but only a few were trialled in humans, including CardiAQ®, Tiara®, Tendyne®, and FORTIS®^[11]^.

CardiAQ® is a symmetrical self-expanding bioprosthesis which anchors on the patient’s mitral annulus. It can be deployed transfemorally or transapically by changing the delivery system. The first-generation device was used in 2012 and Sondergaard et al. had trialled the transapical approach on three elderly patients with severe MR, NYHA Class IV, and not fit for Mitraclip^[31]^. All the devices were accurately placed, patients were almost MR-free after the procedure, and no prosthesis-related complications were reported. However, since the devices were only tested on three selected patients in this article, its safety and efficacy cannot be interpreted. In addition, one of the authors is a consultant for CardiAQ® Valve Technologies Incorporation, which can make the case review biased. Another case study by Ussia et al.^[32]^ used the transfemoral approach for CardiAQ®. The patient had multiple comorbidities, including AF, triple bypass, and factor VII deficiency, therefore he was considered to be at high risk for surgical management. The right femoral vein was accessed, and the posterior part of atrial septum was punctured to allow arteriovenous access. The device was deployed successfully. After the catheter delivery system was removed, an iatrogenic tear at the septum with left to right shunt was noticed and sutured. The patient was stable throughout the operation and discharged with no postoperative complications. The NYHA status has changed from Class III before the operation to Class I at one-month follow-up. Again, there is conflict of interest, since the founder and consultants from CardiAQ® Valve Technologies Incorporation had participated in this study.

The Tiara® device was developed by Neovasc Incorporation®. It uses a transapical approach to deliver a self-expanding frame which assembles the mitral annulus. The sheathless device is fixated by radial expansion and ventricular tabs. Two sizes are available at the moment to accommodate any difference in annular dimensions. Cheng performed the procedure on an 80-year-old man with history of ischaemic cardiomyopathy, myocardial infarction, coronary bypass, vascular disease, previous aortic aneurysm repair, and renal disease^[33]^. The device was placed in optimal position and the mean transvalvular gradient was 2 mmHg. CPB was not used and the patient was haemodynamically stable throughout the operation and had an uneventful postoperative outcome. The patient’s NYHA status was Class III prior to operation, but his postoperative status was not mentioned. These case studies have demonstrated that percutaneous devices are feasible in mitral valve surgery. However, as these are relatively new devices, there are no large retrospective studies, let alone randomized controlled trials (RCTs), assessing the safety and efficacy of the procedure. More investigation is needed to ascertain the benefits in adopting these devices and will minimize the risk of recalling future devices and prevent unnecessary reoperations on patients.

### Conventional Left Atrial or Transseptal Approaches

In order to operate on mitral valves, surgeons typically access them through the LA or TS approach via the right atrium (RA). This method is performed either through a median sternotomy or right thoracotomy^[34]^. While variations exist, atriotomy is performed behind and parallel to the interatrial sulcus^[35]^. The cut is then extended to reach the roof of LA to expose the mitral annulus. A commonly experienced limitation of LA is poor mitral valve visualization, and this is particularly common in patients with deep chest, small LA, or presence of pericardial adhesions. The TS approach is able to offer superior visualization and overcome limitations of the LA approach. TS approach is performed similarly to LA approach, and begins with sternotomy, vena cava cannulation, CPB, and cardioplegia^[36]^. This is followed by opening RA on its anterolateral aspect, from the base of the right atrial appendage to the superior portion of the interatrial septum. The mitral valve is then exposed by an incision from the base of the ascending aorta, along with the interatrial septum, and to the inferior end of fossa ovale. Despite theorized benefits, both approaches are still widely used.

The present literature to support either approach as superior is not strong and has not provided a general consensus; sample sizes and statistical significance vary greatly between studies. In a study by Mujtaba and Clark, of 1,017 patients, the LA cohort had a statistically significant increase in transient ischemic attack (TIA) and strokes when compared to the TS group (94 *vs*. 6; *P*=0.05), respectively^[36]^. AF and heart block, other common postoperative complications, did not differ between the approaches (*P*=0.22 and *P*=0.14), respectively. The ACx and CPB times were similar and did not differ significantly between both cohorts (*P*>0.10). The 30-day mortality did not differ either, with 3.7% in TS group and 4.3% in the LA group (*P*=0.75). TS approach was demonstrated to potentially reduce TIA and stroke risk without affecting ACx or CPB time. Similarly, a TS-LA approaches comparison by Masiello et al.^[37]^ observed no technique-related mortality, but noticed slight increases in ischaemia time and surgical bleeding. In a study by Rezahosseini et al.^[38]^, OR of AF was calculated to be 1.539 (95% CI 1.072-2.210; *P*=0.019), while the mortality rates between the TS and LA approaches did not differ (*P*=0.274), their pump time (160 *vs*. 107 minutes, respectively; *P*<0.001) and ACx time (90 *vs*. 61 minutes, respectively; *P*<0.001) were significantly longer in the TS group than in the LA group.

Another study comparing TS and LA approaches, by Nienaber and Glower, observed a significant increase in patients requiring new pacemakers in the TS group (10.5% *vs*. 5.1%, respectively; *P*=0.025) or the presence of a new junctional rhythm (8.7% *vs*. 4.2%, respectively; *P*=0.035), despite no significant differences in ACx or CPB times^[39]^. Their study included many patients who had concomitant procedures during the operation and had overall longer CPB and ACx times than patients who had isolated mitral procedures, 240 *vs*. 197 minutes (*P*<0.0001) and 150 *vs*. 111 minutes (*P*<0.0001), respectively. However, they reported that the ACx or CPB time of isolated mitral procedures did not differ between TS and LA approaches. Other studies of mitral valve operations with concomitant procedures also observed similar effects: a study of 146 patients by Gaudino et al.^[40]^ identified significantly higher ACx or CPB time, as well as increased AF incidence (60.9 *vs*. 54.0%), in the TS group than in the LA group. Despite available evidence, differences in morbidities between TS and LA approaches and their relationship with varying ACx or CPB time remain unclear. Various TS approaches are known to provide superior visualization of mitral valves; enough to some surgeons consider that its technical advantages outweigh potential arrhythmia risks^[41]^. While the consensus is that both TS and LA approaches are relatively safe, there is a lack of literature to provide a quality comparison. As with many of the studies, much of the observed differences in mortality and morbidity may be attributed to selection bias or concomitant procedures, hence, large randomized trials are needed to identify the superior technique.

Table 4 summarizes the important findings for eight studies which compared TS and LA approaches.

**Table 4:** Summary of studies comparing transeptal (TS) and left atrial (LA) approaches.

Author	Population (n)		Mortality rates (%)		AF incidence (%)		ACx time (minutes)		CPB time (minutes)		Important findings
	TS	LA	TS	LA	TS	LA	TS	LA	TS	LA	
Mujtaba and Clark^[36]^	135	882	3.7	4.3	42.17	35.11	82	78	107	114	TS approach provides improved exposure of mitral valve without significant increase in CPB time, ACx time, or comorbidities
Rezahosseini et al.^[38]^	163	652	6.1	4.1	0.6	0.5	84*	58	90	61	TS approach is associated with increased CPB time, ACx time, and postoperative AF incidence, but not mortality
Légaré et al.^[34]^	18	43	6	7	N/A	N/A	132±32	144±57	177±46	198±86	LA approach increases CPB time, but outcomes are similar
Kumar et al.^[35]^	65	24	4.6	0	26.2**	20.8**	N/A	N/A	101.5	65.5	TS approach provides technical advantage, but may cause junctional transient rhythm via injury to sinus node artery or conduction pathway
Aydin et al.^[41]^	47	44	8.7	4.5	N/A	N/A	96.0±26.9	83.4±43.1	128.3±36.2	118.3±56.8	Superior TS approach is not associated with serious adverse effects compared to LA
Nienaber et al.^[39]^	258	273	1.2	1.5	35***	39***	150	111	240	197	Isolated mini-TS approach does not affect CPB or ACx time
Gaudino et al.^[40]^	73	73	9.6	8.2	54.6	64.0	97.3±35	77±30	79±29	58±20	Superior TS approach is not associated with greater incidence of AF or complications
Masiello et al.^[37]^	110	62	2.7	2	N/A	N/A	65.9±17	67.9±20	N/A	N/A	Technical advantages in TS approach outweigh its minimally increased ischaemia time and minor surgical bleeding

ACx=aortic cross-clamp; AF=atrial fibrillation; CPB=cardiopulmonary bypass

* Results from pure mitral valve surgeries are used.

** Preoperative AF rates are 54.2% and 43.1% for TS and LA, respectively.

*** Preoperative AF rates are 34% and 40% for TS and LA, respectively; no statistically significant differences measured.

## SUMMARY

With persisting research in the field of valvular surgery, techniques in cardiothoracic surgery have been constantly improving. Each step of the surgery, whether it is accessing the mediastinum or the mitral valve itself, has been greatly improved upon. While these developments show great potential, they now warrant large randomized trials to ascertain their benefits before becoming the mainstay treatment approaches. Currently the greatest potential lies within the minimally invasive and robot-assisted approaches, which are generally associated with reduced comorbidities and quicker recoveries. These approaches offer substantially reduced trauma and warrant consideration as a replacement to the traditional sternotomy. Similarly, the percutaneous approach may be able to offer even less trauma, but much more research is needed to compare its benefits with those from other methods. Despite their observed benefits, a common limitation to these approaches lies in the quality of evidence; most literature is confined to retrospective studies, and randomized trials are rare. Some authors have suggested that effects may be caused by confounding factors and patient selection bias. To move forward, the current evidence needs to be further supported by randomized trials prior to becoming the mainstay approaches. All-in-all, modern advancements in surgical techniques and materials may soon allow us to deliver reduced trauma and better outcomes than what mitral valve surgeries have historically offered.

## CONCLUSION

Currently, evidence suggests that MVr tend to provide satisfactory long-term survival in both degenerative and ischaemic mitral valve regurgitation. The traditional approach to access the mediastinum via sternotomy may soon be surpassed by advancements in minimally invasive and robot-assisted approaches, which generally have lower risk of stroke or AF incidences despite longer ACx or CPB times. Alternatively, percutaneous valve surgeries showed some potential in selected patients, but the current literature is limited to case studies and it requires much more research to substantiate its benefits before becoming a mainstay approach.

**Table:** 

Author's roles & responsibilities	
AH	Substantial contributions to the conception or design of the work; or the acquisition, analysis, or interpretation of data for the work; final approval of the version to be published
HTK	Substantial contributions to the conception or design of the work; or the acquisition, analysis, or interpretation of data for the work; final approval of the version to be published
KSF	Substantial contributions to the conception or design of the work; or the acquisition, analysis, or interpretation of data for the work; final approval of the version to be published
